# Investigations of the Nucleating Agent Effects on Polypropylene of Pumice from Three Distinct Areas in Türkiye

**DOI:** 10.3390/polym17212928

**Published:** 2025-10-31

**Authors:** Yasin Özdemir, Metehan Atagur, İbrahim Şen, Kutlay Sever

**Affiliations:** 1 Mechanical Engineering Department, Izmir Kâtip Celebi University, 35620 Izmir, Turkey; kutlaysever@gmail.com; 2 Metalurgical and Materials Engineering Department, Izmir Kâtip Celebi University, 35620 Izmir, Turkey; metehan26mmz@gmail.com; 3 Central Research Laboratory, Bursa Technical University, 16310 Bursa, Turkey; ibrahim.sen@btu.edu.tr

**Keywords:** pumice, nucleating agent, polypropylene (PP)

## Abstract

This study investigates the mechanical and thermal properties of polypropylene (PP) composites incorporating pumice, a naturally occurring porous volcanic rock with high SiO_2_ content, sourced from three regions in Türkiye (Nevşehir, Alaçatı, and Kütahya). Pumice was processed to particle sizes below 10 microns to maximize nucleating effectiveness, and composites were fabricated by melt compounding. The distinct mineralogical composition, porosity, and surface chemistry of the pumice samples enabled systematic evaluation of how regional variations influence crystallization and mechanical performance. A multi-analytical characterization approach, including thermogravimetric analysis (TGA), differential scanning calorimetry (DSC), X-ray diffraction (XRD), and standardized mechanical tests (tensile, flexural, and impact), was applied. Results revealed that Alaçatı pumice at 0.1 wt% increased the impact strength of PP by about 11%, while maintaining stiffness. This demonstrates that pumice, unlike conventional fillers, can simultaneously enhance toughness and rigidity. Thermal analysis confirmed improved stability, with higher degradation onset and maximum decomposition temperatures observed in pumice-filled PP. DSC results indicated that certain pumice loadings promoted nucleation and increased crystallinity, while excessive amounts disrupted chain packing. SEM examinations confirmed uniform dispersion at low loadings, with agglomeration at higher levels reducing impact resistance. This work provides the first systematic demonstration of pumice powders as effective nucleating agents in PP, combining regional mineralogical diversity with measurable performance benefits. These findings indicate that pumice can serve as a sustainable, low-cost alternative to conventional nucleating agents, with potential applications in polymer components requiring improved toughness and thermal stability.

## 1. Introduction

Engineering material advancements drive technological innovation. Polymer composites have become increasingly common in recent years due to their numerous advantages over traditional materials. These lightweight composites offer significant benefits across various industries, including rail, automotive, and aerospace, particularly through reduced fuel consumption [1]. In recent decades, the polymer industry has shown growing interest in the use of fillers. The incorporation of fillers significantly enhances polymer properties, including flexibility, impact resistance, tensile strength, abrasion resistance, and thermal stability [2,3,4]. Various fillers, including pozzolanic wastes, mineral fillers, and agricultural wastes, have been employed to achieve desired material properties. Researchers have extensively studied how filler characteristics—including type, size, content ratio, and filler-matrix adhesion influence the mechanical properties of polymer composites [5].

Polypropylene (PP), a versatile thermoplastic polymer, finds extensive applications due to its advantageous properties, including low cost, low density, excellent processability, and recyclability [6]. However, its limited fracture toughness presents challenges in specific applications. One effective approach to overcome PP’s limitations involves using heterophasic ethylene–propylene copolymers (EPP), comprising a semi-crystalline isotactic PP homopolymer matrix with a dispersed soft ethylene–propylene rubber (EPR) phase [7]. While the strategically distributed elastomeric phase enhances toughness and low-temperature impact resistance, it adversely affects material rigidity [8]. Therefore, to achieve an optimal balance between toughness and stiffness, researchers have modified the polymeric matrix using moderate amounts of hard nanoparticles [9].

Orthorhombic (γ), hexagonal (β), and monoclinic (α) crystal modifications are all present in PP, a semicrystalline and polymorphic material. The low impact resistance of PP limits its potential as an engineering polymer, even though it has outstanding ductility and stiffness, excellent resistance to chemicals and moisture, and better processability. Although PP toughening by adding tiny particles, including ethylene–propylene rubber and polyethylene-octene elastomer copolymer, has been the subject of many investigations, the rubber toughening method usually reduces the modulus and strength of PP. In order to simultaneously improve strength and toughness, recent research has concentrated on ternary systems that combine rubber with hard inorganic fillers (such as talc, calcium carbonate and silica). However, mismatching parameters like component miscibility, phase structure, and phase size can significantly impact the composite’s mechanical properties. Notably, β-form PP (β-PP), produced using specific β-nucleating agents (β-NA), demonstrates superior impact strength and heat distortion temperature compared to α-form PP (α-PP) [10,11,12].

Nucleating agents, when incorporated into plastics, form nuclei for crystal growth in polymer melts. In PP, these agents enhance crystallinity and create more uniform crystalline structures through additives such as adipic and benzoic acids or their metal salts. They usually decrease processing times while improving physical characteristics and, in the case of α-NAs (clarifying agents), improving aesthetics. PP nucleating compounds fall into two categories: α-nucleating agents inducing α-phase crystallization and β-nucleating agents promoting trigonal β-phase formation. Common α-NAs include talc, kaolin, and sodium benzoate, while β-NAs comprise substances like pimelic acid with calcium stearate and triphenodithiazine [13,14].

The impact of pumice as a nucleating agent on the mechanical and thermal properties of PP is examined in this work. During rapid lava cooling, dissolved gas precipitation gives pumice, an amorphous, porous volcanic rock mostly made of SiO_2_, its distinctive porous structure [15]. Türkiye, possessing nearly 3 billion m^3^ of reserves, ranks as the world’s fifth-largest pumice producer after Italy, Greece, China, and Iran [16]. The material exists in two forms—acidic and basic pumice—with acidic pumice being predominant both in Türkiye and globally. Pumice’s porous surface shape and changeable acidity (acidic/basic character) can significantly affect filler–matrix interactions and crystallization behavior in PP, making it especially appropriate for polymer reinforcement in addition to its availability [17].

Pumice is useful in construction applications, especially in lightweight brick and concrete parts that provide thermal and acoustic insulation, because of its high porosity, which leads to a low apparent density (0.35–0.65 g/cm^3^) in aggregate form [18]. Studies have looked into pumice’s potential as an adsorbent for both organic (such as phenol and textile colors) and inorganic (such as various heavy metals and radioactive elements) water contaminants, expanding its usefulness beyond its usual applications [19,20,21,22,23].

Despite these applications, the use of pumice as a nucleating agent in PP has rarely been addressed in the literatüre [24], making it a novel low-cost alternative compared with traditional nucleating agents like talc or silica [25]. Compared to other natural nucleating agents such as talc and silica, pumice offers unique advantages in terms of abundance, low cost, and a highly porous surface morphology that can facilitate mechanical interlocking with the polymer matrix. Moreover, unlike talc, which has raised environmental and health concerns regarding occupational exposure (e.g., respirable dust), pumice is generally considered a safer and more sustainable alternative [25].

Furthermore, pumice deposits differ by region in mineralogical composition, porosity, and surface chemistry [26,27,28], which are critical factors for nucleation [29]. Compared to other natural nucleating agents such as talc, silica, and zeolite, pumice offers unique advantages in terms of abundance, low cost, and porous surface morphology. Moreover, unlike talc, which has raised environmental and health concerns in occupational exposure, pumice is considered safer and more sustainable.

Although talc, silica, and zeolite have been widely studied as nucleating agents, most of these are synthetic or relatively costly, while pumice represents an abundant and sustainable natural resource [25]. To date, however, a systematic comparison of pumice from different geographical regions in terms of nucleation efficiency has not been reported [28]. The mineralogical characteristics of pumice, including silica content, porosity, and acidic/basic nature, are critical parameters that directly influence filler–matrix interactions and nucleation. For instance, acidic pumice with higher SiO_2_ content may promote stronger interactions and faster nucleation, while basic pumice, containing more alkaline oxides, tends to weaken interfacial bonding. Therefore, understanding regional mineralogical differences is essential to evaluate pumice as a functional nucleating agent in PP.

Therefore, the central research question of this study is how regional variations in pumice mineralogy, surface chemistry, and particle size influence nucleation behavior, crystallization kinetics, and the mechanical/thermal properties of PP composites.

By employing pumice from three distinct regions in Türkiye (Nevşehir, Alaçatı, and Kütahya), this work aims to fill this gap and provide the first comprehensive assessment of natural pumice as a functional nucleating agent in PP [24,30,31].

## 2. Experimental Details

### 2.1. Materials

**Figure 1:**
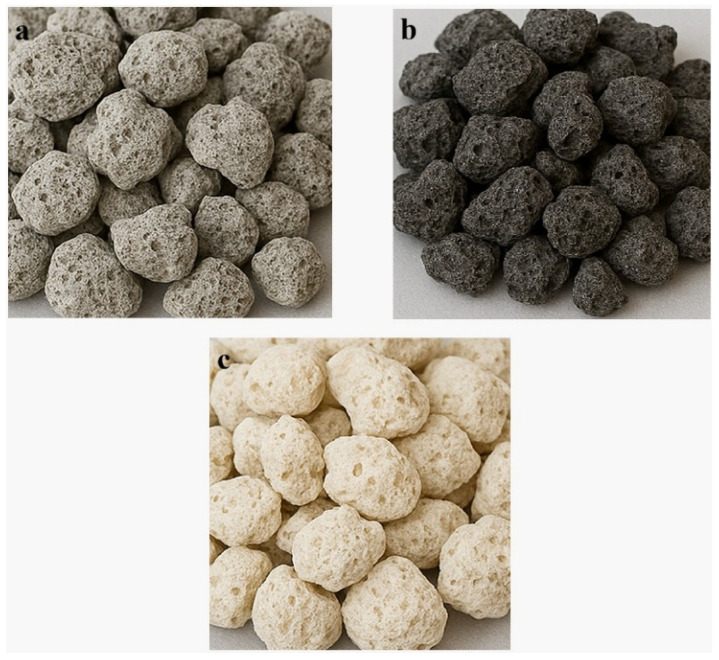
The pumice samples were obtained from three regions in Türkiye (**a**) Nevşehir, (**b**) Kütahya, and (**c**) Alaçatı.

The heterophasic polypropylene copolymer Moplen 2000 HEXP (T_m_ = 165 °C, ρ = 0.9 g/cm^3^, melt flow rate (230 °C/2.16 kg) = 16 g/10 min), manufactured by Lyondell Basell Company, was used as the polymeric matrix material. The heterophasic polypropylene copolymer (Moplen 2000 HEXP) consists of a semi-crystalline isotactic PP homopolymer matrix with a dispersed amorphous ethylene–propylene rubber (EPR) phase. This structure classifies it as a block-type heterophasic copolymer, where the EPR phase is polymerized within the PP matrix to improve impact strength. The typical ethylene content in such impact copolymers ranges from 5–15% by weight, which enhances toughness without significantly compromising stiffness [7]. The pumice samples were obtained from three regions in Türkiye: Nevşehir, Alaçatı-İzmir, and Kütahya (Figure 1). The technical properties and chemical compositions of the pumice samples are presented in Table 1 and Table 2, respectively [32]. It is noteworthy that while the initial characterization highlighted Alaçatı pumice for its high silica content, the chemical composition data (Table 2) reveals that Nevşehir pumice actually possesses the highest SiO_2_ content (70.85%), followed by Alaçatı (64.33%) and Kütahya (61.22%). This mineralogical distinction is crucial as it directly influences the nucleation efficiency and interfacial interactions with the PP matrix, which will be discussed in the subsequent sections.

The chemical compositions of the three pumice types (Table 2) show significant variations. Notably, the SiO_2_ content varies by approximately 9.6% between Nevşehir (highest) and Kütahya (lowest). Conversely, the alkaline oxide content (Na_2_O, K_2_O, CaO) is lowest in Nevşehir and highest in Kütahya pumice. These substantial differences in composition are expected to significantly affect their surface chemistry (acidic/basic character) and, consequently, their nucleating efficiency and interfacial adhesion with the polypropylene matrix [26,27]. Because of their varied mineralogical compositions and widespread industrial availability, three unique sources of pumice, Nevşehir, Alaçatı, and Kütahya, were chosen for this investigation. Kütahya pumice is distinguished by its intermediate composition with significant alkaline oxides, Alaçatı pumice by its high silica content and crystalline phases, and Nevşehir pumice by its comparatively high porosity and amorphous structure. A methodical study of the effects of regional differences in surface chemistry and mineralogy on nucleation efficiency in polypropylene composites was made possible by this selection.

### 2.2. Preparation of Materials for Extrusion Process

**Figure 2:**
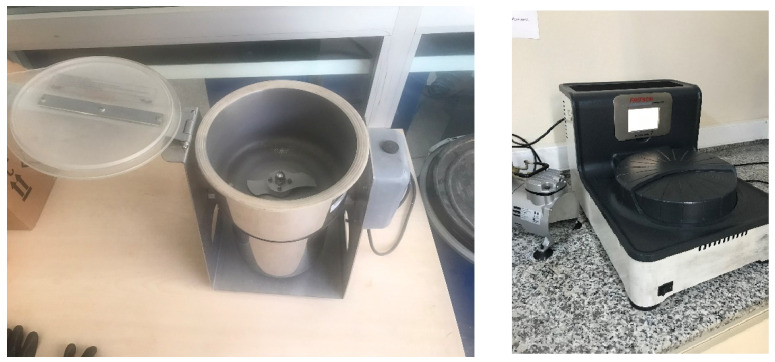
Grinding devices.

The preparation of pumice involved a two-stage grinding process. In the first stage, raw pumice was ground to particles smaller than 100 microns using a bladed super mixer, followed by sieving. Based on previous research indicating enhanced nucleating agent effects at smaller particle sizes, the sieved pumice (<100 µm) was further processed using a Fritsch Pulverisette 7 ball mill at 400 rpm for 4 h using zirconia balls (10 mm diameter) with a ball-to-powder ratio of 10:1 to achieve particle sizes below 10 microns. The variation in the final average particle sizes) among the different pumice sources can be attributed not only to the consistent grinding parameters applied to all samples but also to their inherent mineralogical hardness and structural characteristics [27,28]. For instance, the more crystalline Alaçatı pumice yielded finer particles compared to the more amorphous and porous Nevşehir pumice. The grinding equipment setup is illustrated in Figure 2.

### 2.3. Production of PP Composites

PP composites were manufactured using a twin-screw extruder (GÜLNAR PLASTİK MAKİNALARI SAN. VE TİC. LTD. ŞTİ.- Kayseri, Türkiye) with an L/D ratio of 40 and screw diameter of 16 mm. Prior to extrusion, PP granules and pumice samples were physically premixed. The material processing involved a five-zone temperature profile: 160 °C (Zone 1), 175 °C (Zone 2), 170 °C (Zone 3), 160 °C (Zone 4), and 135 °C (die zone), with a screw speed of 200 rpm. The extruded filaments were water-cooled and subsequently granulated. To eliminate residual moisture, the granules were dried in an oven at 100 ± 2 °C for 24 h. The sample nomenclature and composition ratios are detailed in Table 3. In this table, regional pumice sources are shown by their initials, and numerical prefixes indicate the loading levels. The pumice content was limited to a maximum of 0.3 wt% based on preliminary experiments and literature findings [24], which indicated that while low loadings of mineral fillers can act as effective nucleating agents, higher concentrations often lead to particle agglomeration (as confirmed by our SEM results), which acts as stress concentrators and deteriorates mechanical properties, particularly impact strength. This trend is clearly observed in our results, where impact strength generally decreased at 0.3% loading compared to 0.1%.

In order to eliminate the moisture content, the PP composites were baked at 80 °C for two hours before being injected. An injection molding machine (BOY/22A) with a screw diameter of 40 mm and a clamping force of 220 kN was used to create tensile, flexural, and impact test specimens.With a cooling period of 25 s, the cycle time was roughly 40 s. Between zones 1 and 4, the process temperatures were set at 195, 205, 200, and 185 °C. Injection-molded samples were used for all characterization experiments.

## 3. Characterization

### 3.1. Particle Size Analysis

Particle size distributions were measured using two different instruments: a Malvern Panalytical Ltd., Newland, UK, Master Sizer Hydro 3000 for the initial ground pumice (<100 microns) and a Malvern Panalytical Ltd., Newland, UK, Nano ZS for the fine particles (<10 microns) obtained after secondary grinding. In applications where granules are implemented, particle size is among the most critical attributes. Particle sizes in dry and wet samples can be measured using the Malvern Nano ZS instrument from the nanometer to the millimeter range.

### 3.2. Thermogravimetric Analysis (TGA)

Using a TA Instrument Q600, New Castle, DE, USA the thermal decomposition characteristics of PP and its composites were assessed. Under a nitrogen environment, samples were heated from ambient temperature to 600 °C at a rate of 10 °C per minute.

### 3.3. Differential Scanning Calorimetry (DSC)

A TA Instruments DSC Q2000, New Castle, DE, USA in a nitrogen environment was used to characterize the thermal characteristics. Heating from 20 °C to 200 °C at 10 °C/min, isothermal holding at 200 °C for 3 min to remove thermal history, and cooling to 20 °C at 10 °C/min, followed by a second heating cycle to 200 °C at 10 °C/min, were the three processes making up the analysis. These measurements were used to calculate the melting and crystallization temperatures as well as the corresponding enthalpy values.

### 3.4. Heat Deflection Temperature (HDT)

HDT tests were performed on an Instron, Norwood, MA, USA, Ceast HV3 model device standard under a specific load of 1.8 MPa.

### 3.5. X-Ray Diffraction (XRD)

A Bruker, Billerica, MA, USA, D8 Advance Diffractometer fitted with a Ni-filtered CuKα radiation (λ = 1.54 Å) was used to obtain the XRD patterns. Every sample was measured in the 2θ angle range of 5° to 50°. The scan step time was 10 s, and the step size was 0.03 s. A voltage of 45 kV and 10 mA of current were utilized.

### 3.6. Mechanical Properties

Tensile properties were evaluated according to ISO 527 using a Shimadzu, Koyoto, Japan, Autograph AG-IS universal testing machine equipped with a 5-kN load cell at a crosshead speed of 50 mm/min. Flexural properties were determined following ISO 178 using three-point bending tests with a span length of 64 mm and a crosshead speed of 1 mm/min. Impact resistance was assessed using an Instron, Norwood, USA Ceast 9050 impact tester with a 5.5 J Izod hammer. All mechanical tests were performed on five specimens per composition (*n* = 5), and the results are reported as average values with standard deviations [33].

### 3.7. Scanning Electron Microscopy (SEM)

The composites’ fracture surfaces were inspected with a Carl Zeiss, Oberkochen, Germany, 300VP scanning electron microscope set to 2.5 kV. To avoid charging effects, samples were covered with a thin layer of gold using an Emitech, Trappes, France, K550X automatic sputter coater before being observed.

Scanning electron microscopy (SEM) was used to investigate the fracture surfaces of tensile and impact specimens. Special attention was given to filler dispersion, agglomeration size, and interfacial voids in order to understand the interaction between pumice particles and the PP matrix.

## 4. Results and Discussion

### 4.1. Particle Size Analysis

Using dynamic light scattering (DLS) analysis, the particle size distributions of pumice samples from three distinct geographic regions were characterized. Table 4 presents the average particle sizes and the polydispersity index (PDI) of the ground pumice samples after the secondary milling process. The Alaçatı pumice exhibited the smallest average particle diameter of 549.2 nm with a relatively narrow distribution (PDI: 0.21), followed by the Kütahya sample at 630.5 nm (PDI: 0.25). The Nevşehir pumice showed the largest average particle size of 904.3 nm (PDI: 0.28), approximately 1.65 times larger than the Alaçatı sample. The low PDI values indicate a reasonably uniform particle size distribution achieved after ball milling, which is crucial for consistent nucleation behavior. This variation in particle sizes among different geographical sources can be attributed to the inherent mineralogical differences and structural characteristics of the pumice deposits, as well as their response to the grinding process.

These dimensions are particularly significant for the intended application as nucleating agents in polymer composites, as particles in the sub-micron range can provide enhanced surface area for nucleation while maintaining good dispensability within the polymer matrix. Moreover, the differences in average particle sizes among the samples may influence their effectiveness as nucleating agents in the PP matrix, as smaller particles typically provide more nucleation sites per unit volume. Because of its small size and high specific surface area, the particle can supply additional nucleation sites and raise the probability of nucleation at high temperatures [34].

### 4.2. Mechanical Properties

As a result of incorporating pumice from different geographical regions of Türkiye into the polypropylene (PP) matrix, changes in tensile, flexural and impact properties were investigated depending on both the pumice source and concentration. For micro- and nano-particulate composites, the mechanical properties depend on the effectiveness of stress transfer between the matrix and the fillers. Key factors influencing the mechanical strength of the composite include particle size, particle/matrix interface, and particle loading. Fillers with irregular shapes also do not contribute effectively to stress transfer to the matrix under tensile load [35]. The sharp points of these irregularly shaped fillers result in stress concentration within the polymer composites [36,37].

**Figure 3:**
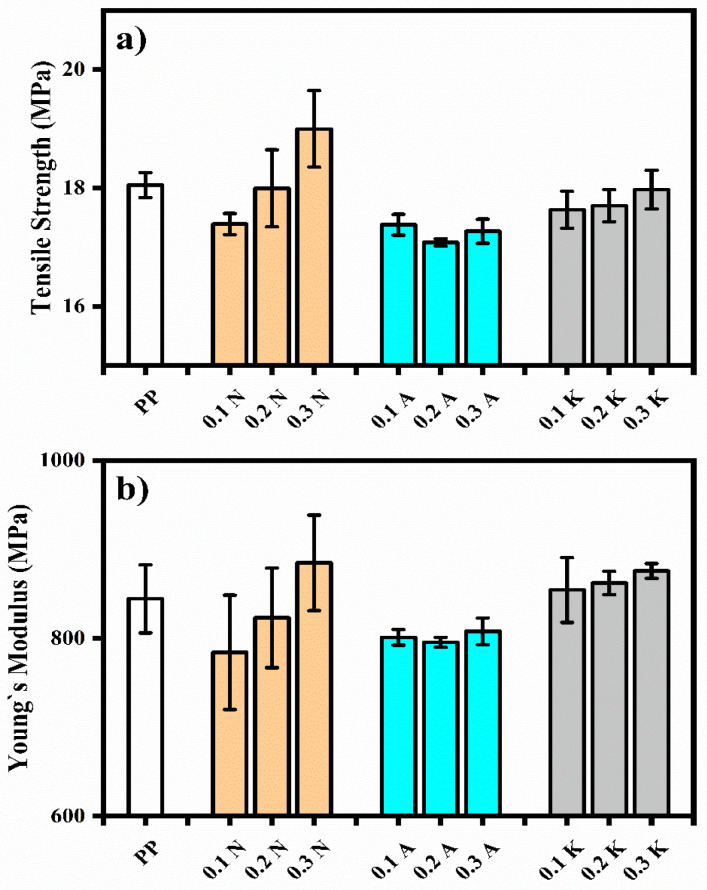
(**a**) Tensile strength and (**b**) Young’s Modulus of of PP and Its Composite.

Table 5 presents the mechanical test results of pure PP and PP composites. In this study, the mechanical performance of the composites shows a complex relationship between the filler content and tensile strength. At 0.3 wt% loading, Nevşehir pumice showed the most significant increase in tensile strength, from 18.05 ± 0.21 MPa (neat PP) to 18.99 ± 0.64 MPa, representing an increase of 5.2% (Figure 2). This increase can be attributed to the effective stress transfer between the polymer matrix and the pumice particles. However, this was different compared to other pumice sources; Alaçatı and Kütahya pumice samples showed a slight decrease in tensile strength. In the 0.3N sample, the Young’s modulus value increased by the greatest amount (4.79%), reaching 884.96 ± 53.68 MPa when compared to pure PP (Figure 3). This increase in stiffness indicates that the pumice particles have effectively reinforced the polymer matrix, most likely as a result of their rigidity and good dispersion at lower concentrations.

**Figure 4:**
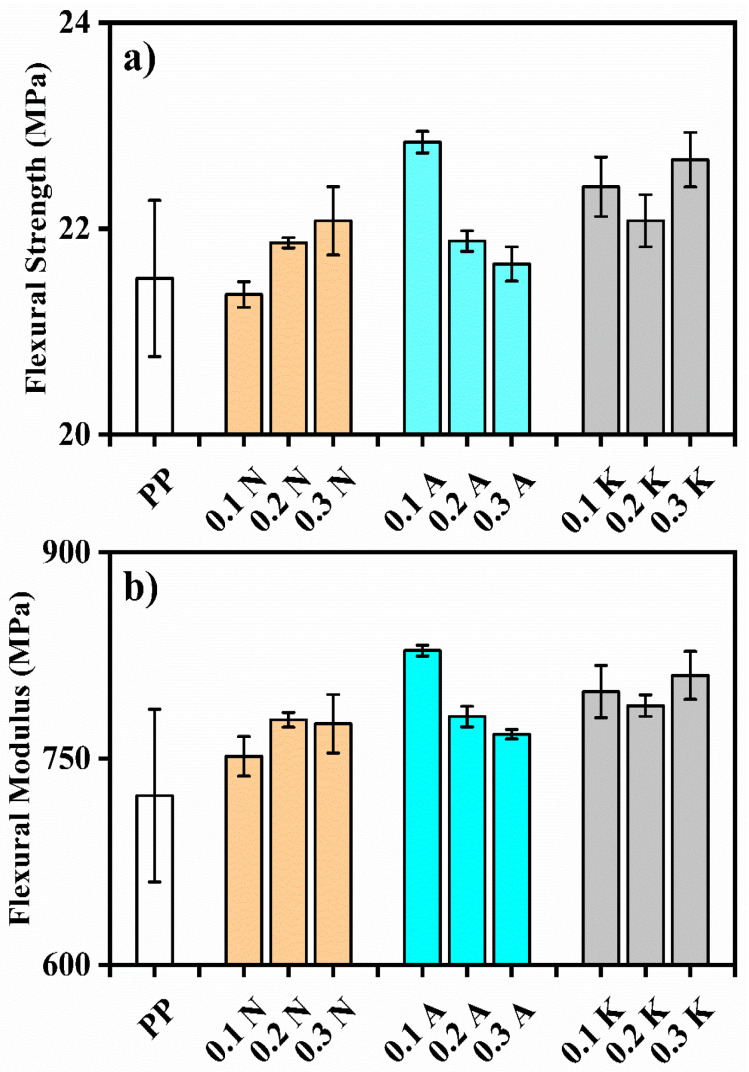
(**a**) Flexural strength and (**b**) Flexural Modulus of of PP and Its Composite.

Flexural properties showed moderate but consistent improvements, with the 0.1A sample achieving the highest flexural strength of 22.84 ± 0.1 MPa compared to neat PP’s 21.51 ± 0.75 MPa (6.2% increase) (Figure 4). Notably, Kütahya pumice samples demonstrated consistent enhancement in flexural strength across all concentrations (22.08–22.67 MPa), suggesting uniform distribution and good interfacial adhesion between the filler and matrix.

**Figure 5:**
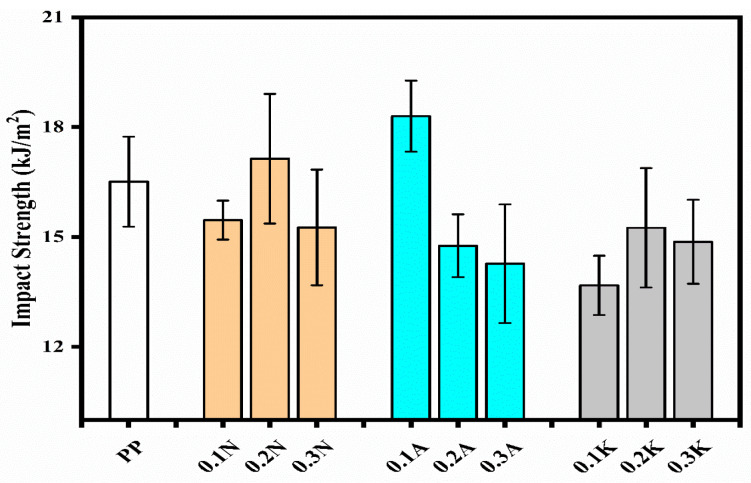
Impact Strength of PP and PP Composite.

Impact strength results revealed an interesting trend where optimal performance was achieved at lower filler concentrations. The 0.1A sample showed the highest impact strength of 18.30 ± 0.97 kJ/m^2^, surpassing neat PP’s 16.51 ± 1.23 kJ/m^2^ by 10.8% (Figure 5). However, higher filler loadings generally led to decreased impact strength, possibly due to increased brittleness and potential agglomeration of pumice particles at higher concentrations. The observed improvement in impact strength at low pumice loadings indicates that pumice-filled PP composites could be useful in applications requiring toughness and durability. Potential sectors include automotive interiors (e.g., dashboards, door panels), household appliances, and packaging, where lightweight design, recyclability, and cost-effectiveness are critical.

This comprehensive review reveals that pumice can effectively enhance the mechanical properties of PP composites and that the performance is highly dependent on both source and concentration. The results indicate that careful selection of pumice source and concentration is crucial to target specific property improvements in PP composites [38]. In particular, further investigation of pumice particle morphology and surface properties may provide valuable insights for future composite development. To further elucidate the role of particle size, the specific mechanical properties (e.g., impact strength per unit surface area of filler) could be considered. However, the observed trends cannot be explained by particle size alone. For example, Alaçatı pumice, with the smallest particle size, provided the highest impact strength at 0.1 wt%, whereas Kütahya pumice, with an intermediate size, showed lower impact values. This underscores that the mineralogical composition and surface chemistry of the pumice are dominant factors over mere particle size in determining mechanical performance.

### 4.3. Thermogravimetric Analysis (TGA)

**Figure 6:**
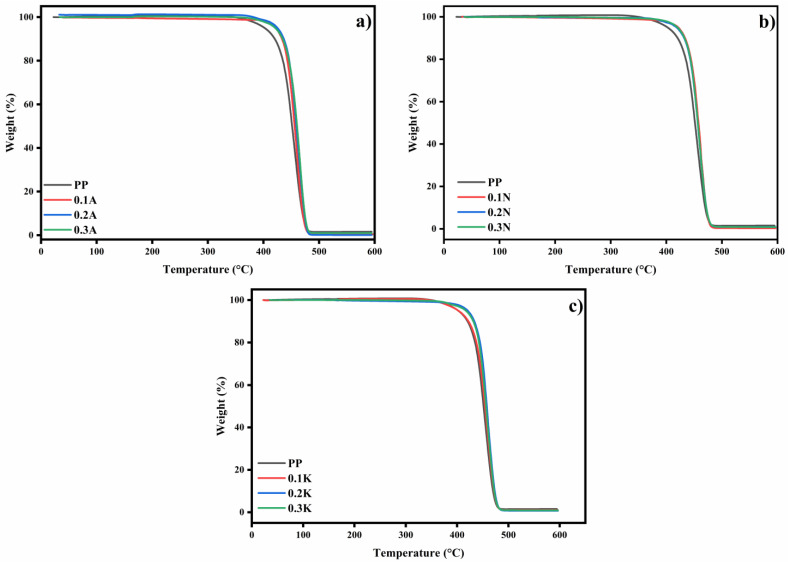
TGA thermograms of (**a**) Alaçatı pumice filled PP composites, (**b**) Nevşehir pumice filled PP composites, and (**c**) Kütahya pumice filled PP composites.

Thermogravimetric analysis was performed to evaluate the thermal decomposition behavior of pumice-filled polypropylene (PP) composites obtained from three different geographical locations (Kütahya, Alaçatı and Nevşehir). In Figure 6, the thermal degradation behavior of PP and its composites is shown. While pure PP had a T_d_ of 402.2 °C at 5% mass loss and a maximum decomposition temperature (T_max_) of 456.23 °C, the incorporation of Kütahya pumice at 0.2 wt% into PP showed improved thermal stability (T_d_ of 420.06 °C at 5% mass loss and a T_max_ of 459.08 °C). It was observed that Alaçatı pumice provided significant improvements in thermal stability. With the addition of 0.1, 0.2, and 0.3 wt% Alaçatı pumice to PP, T_d_ values were obtained as T_d_ = 421.33 °C, 420.42 °C and 420.5 °C, respectively. Also, T_max_ values were found as T_max_ = 460.9 °C, 463.43 °C, and 465.05 °C, respectively. Nevşehir pumice-filled PP composites demonstrated enhanced thermal stability. With the addition of 0.1, 0.2, 0.3 wt%, Td = 420.92 °C, 417.98 °C, and 419.44 °C and Tmax = 461.18 °C, 459.06 °C and 460.54. Notably, Alaçatı pumice exhibited the most pronounced effect on mass loss at 600 °C, particularly at lower concentrations, suggesting modified degradation kinetics at elevated temperatures. The improved thermal stability can be attributed to several mechanical factors. Mineral fillers act as physical barriers that inhibit the diffusion of volatile decomposition products and thus increase the decomposition temperature. In addition, improved thermal conductivity facilitates uniform heat distribution and reduces local thermal degradation. The formation of thermally stable carbonized layers further contributes to the improved thermal resistance [39,40]. These findings have important implications for the development of thermally enhanced PP composites for industrial applications. Experimental data have shown that incorporating geographically diverse mineral fillers into PP results in significant improvements in the thermal stability of PP, with the magnitude of the improvement being dependent on both the filler type and concentration [41].

The primary conclusion from the TGA results in Table 6 is that the incorporation of pumice, irrespective of its geographical source, induces a significant retardation in the initiation of thermal decomposition, as evidenced by the consistent increase in T_d (temperature at 5% mass loss). While variations in T_max and final residue were observed, the most substantial and consistent effect across all composites was the enhanced initial thermal stability. This improvement is predominantly attributed to the barrier effect of the dispersed pumice particles, which hinder the diffusion of volatile decomposition products.

### 4.4. Differential Scanning Calorimetry (DSC)

**Figure 7:**
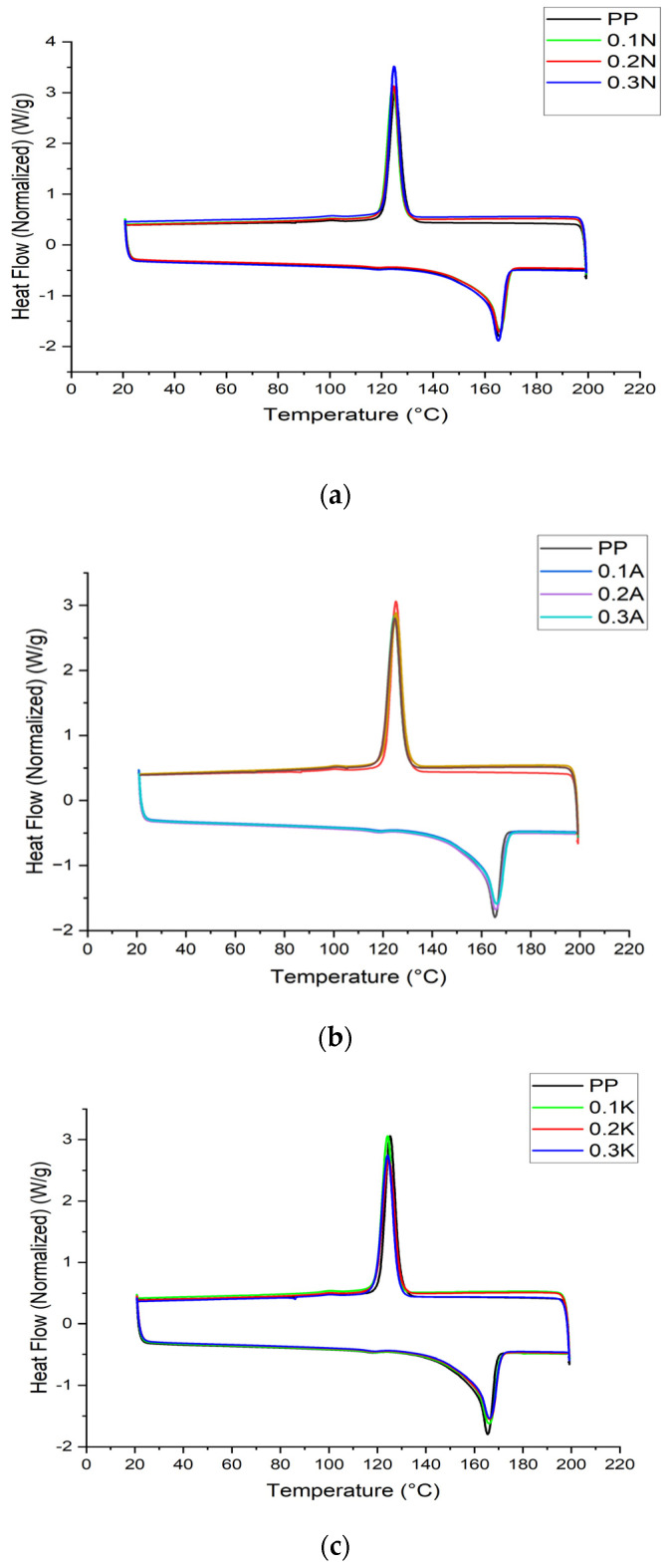
DSC thermograms of (**a**) Nevşehir pumice filled PP composites, (**b**) Alaçatı pumice filled PP composites, and (**c**) Kütahya pumice filled PP composites.

DSC was used to understand the effect of pumice on the crystallization and melting behavior of PP. DSC results are presented in Figure 7. Pumice, a naturally occurring volcanic rock, is known for its porous and mineral-rich composition and can affect the polymer matrix in various ways when used as a filler. DSC results showed that the melting temperatures (T_m_) of all samples were relatively consistent, ranging from 165.24 °C to 166.28 °C, and there was no significant shift in T_m_ due to the presence of pumice. This suggests that pumice did not directly affect the melting temperature of the polymer. The crystallization temperatures (T_c_) were similarly constant, ranging from 124.24 °C to 125.25 °C, indicating that pumice did not significantly alter the crystallization behavior of PP either.

Degree of crystallinity for PP and its composites;

(1) Xc%=ΔHmƟPPΔH°m × 100

Equation (1) was used to calculate the X_c_ values. The expressions in the formula are as follows: H_m_ = melting enthalpy of 100% crystal PP, ϕ_pp_ = weight percentage of PP in the composite. The melting enthalpy of 100% crystalline PP was assumed to be 209 J/g in the X_c_ calculation [41].

However, the heat of fusion (ΔH_m_) and crystallinity (X_c_) values revealed the effects of the inclusion of pumice. The X_c_ value of neat PP was 41.16%. The sample modified with pumice at 0.2A concentration exhibited the highest crystallinity (X_c_ = 40.44%), suggesting that pumice may have a nucleating effect at certain concentrations and promote more regular crystallization in the polymer matrix. This is consistent with previous studies showing that certain fillers, including mineral-based additives such as pumice, can serve as nucleating agents and enhance the formation of crystalline structures by providing sites for polymer chains to align and crystallize more efficiently [42,43,44]. In contrast, samples with higher pumice content, such as 0.3K, showed lower crystallinity (X_c_ = 38.08%), which may be due to the increased amount of pumice particles that disrupt the crystallization process of the polymer. The porous structure of pumice may affect the ability of polymer chains to organize into a crystalline phase, thereby reducing the overall crystallinity. Furthermore, the high surface area of pumice may lead to interactions with the polymer matrix that, depending on the concentration and distribution of the particles, inhibit or alter chain packing. In fact, this decrease in crystallinity suggests that the pumice particles may have disrupted the regular arrangement of the polymer chains and potentially acted as a physical barrier to crystallization [34]. In conclusion, the non-isothermal crystallization data obtained from DSC, particularly the consistent T_c values across composites, suggest that pumice particles act as heterogeneous nucleating sites. Although pumice did not significantly alter the melting temperature of PP, it did affect the crystallization behavior, with certain concentrations acting as nucleating agents and others possibly impairing crystallization. A more detailed investigation into the crystallization kinetics, such as applying the Avrami model to isothermal crystallization data, would provide deeper insights into the nucleation mechanism and crystal growth dimensions. This is proposed as a focus for future work. Further studies on the mechanical and morphological properties of pumice-modified polypropylene will be valuable to understand the full effect of pumice as an additive in polymer processing and its potential applications in areas where thermal and mechanical performance are critical.

These variations highlight the importance of the filler type and concentration in tailoring the thermal and structural properties of PP composites for specific applications [42]. The observed changes are likely due to the physical barrier effect, improved thermal conductivity, and the potential nucleating effect of the mineral fillers, as well as the formation of char layers that protect the polymer matrix during thermal degradation [45,46]. These findings are critical for developing PP composites with tailored properties for enhanced performance in various industrial applications [47]. Table 7 presents the DSC results of pure PP and pumice-added PP composites.

DSC crystallization curves further confirmed the influence of regional pumice variations on nucleation kinetics. Alaçatı pumice yielded the highest crystallization temperature (Tc), with a shift of +X °C compared to neat PP, suggesting that its higher silica content provides efficient nucleation sites. Kütahya pumice showed an intermediate Tc increase, while Nevşehir pumice produced the lowest shift, consistent with its amorphous-rich composition and weaker interfacial bonding. The crystallinity degree (X_c_) followed a similar trend, with Alaçatı pumice promoting the highest overall crystallinity. These results are in agreement with previous studies that reported filler composition as a key determinant of nucleation efficiency in PP composites [36].

DSC thermograms primarily reflected α-form transitions (Tm, Tc), and no distinct β-crystallization peaks were detected. This limitation indicates that DSC alone may not be sufficient to resolve β-phase content in PP/pumice composites, as β-PP formation is better identified by XRD analysis of the 2θ ≈ 16.1–21.8° region [48]. Hence, the β-nucleation effect of pumice was primarily inferred from XRD rather than DSC.

### 4.5. Heat Deflection Temperature (HDT)

In this study, the effect of various pumice powders as mineral fillers on the HDT of polypropylene (PP) was investigated. The baseline HDT of pure PP was found to be 45.23 °C ± 0.67. When pumice powders were incorporated into the PP matrix, significant improvements in HDT were observed, with variations depending on the type and concentration of the filler. Nevşehir pumice, in particular, resulted in the most substantial increases in HDT, with improvements ranging from 5.29% to 8.03% as the filler content increased from 0.1% to 0.3%. The highest increase in HDT occurred at the 0.3% concentration, suggesting that higher concentrations of Nevşehir pumice were particularly effective in enhancing the thermal stability of the PP. Kütahya pumice also led to notable improvements, with HDT increases between 6.82% and 7.82%, although the greatest enhancement was observed at the lowest concentration (0.1%) (Table 8). On the other hand, Alaçatı pumice exhibited the smallest improvements in HDT, ranging from 2.97% to 3.62%. Additionally, at the highest filler concentration (0.3%), Alaçatı pumice caused a slight decrease in HDT, indicating that higher concentrations may not always lead to thermal performance gains with this particular pumice type. These results demonstrate the general effectiveness of mineral fillers, such as pumice, in enhancing the thermal properties of PP, although the extent of improvement is highly dependent on both the type and concentration of the filler [45]. The relationship between filler concentration and HDT improvement was not linear, suggesting that each pumice type has an optimal concentration for maximizing thermal performance. The thermal enhancement is likely attributed to the inorganic pumice particles acting as reinforcements within the polymer matrix, restricting the movement of polymer chains at elevated temperatures, thus increasing the overall stiffness and thermal stability of the composite [43]. This enhancement in thermal properties could potentially expand the applications of PP in high-temperature environments, providing a more cost-effective alternative to more expensive high-performance plastics [49]. Pumice, being an abundant and inexpensive material, offers a viable option for improving PP’s high-temperature resistance without significantly raising material costs [50]. These findings could lead to the development of more heat-resistant PP composites suitable for a wider range of industrial uses [51].

### 4.6. X-Ray Diffraction Analysis (XRD)

**Figure 8:**
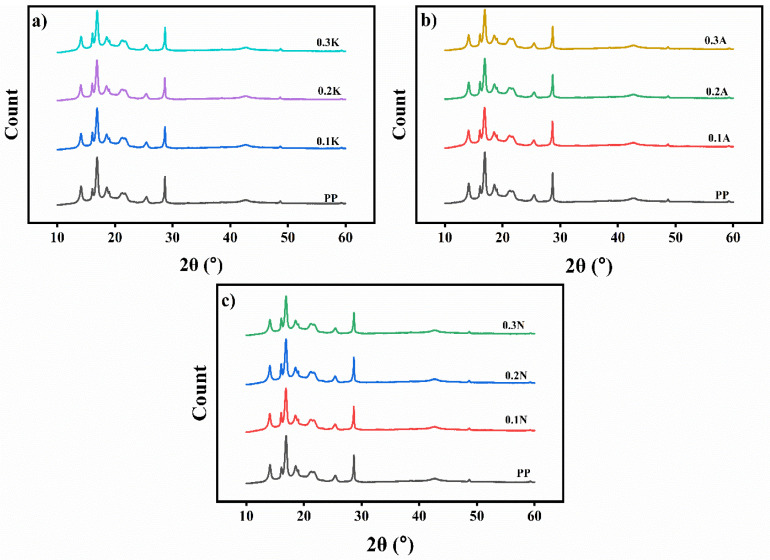
X-Ray Diffractograms (XRD) of (**a**) Kütahya pumice filled PP composites, (**b**) Alaçatı pumice filled PP composites, and (**c**) Nevşehir pumice filled PP composites.

X-ray diffraction analysis of polypropylene with pumice powder from different regions revealed similar findings. XRD results indicate that the characteristic peaks of PP are preserved in all samples. Because of the reflections of the (110), (040), (130), (111), and (131) crystal planes of PP, respectively, PP displays the distinctive diffraction peaks of α-phase at 2θ = 14.1, 16.8, 18.5, 21.1, and 21.8°, as shown in Figure 8. Additionally, the (300) and (301) planes of the β-phase are shown by the distinctive peaks at 2θ = 16.1 and 21.8°, respectively [40].

The study showed that adding any pumice did not change the basic crystal structure of polypropylene. The X-ray patterns remained consistent across all samples, indicating that the pumice particles were well-distributed throughout the material. The combined analysis of XRD, TGA, DSC and HDT data suggests that different pumice powders do not significantly alter PP’s crystalline structure but do affect some thermal properties. This suggests that while the fundamental structure remained the same, the pumice powder still influenced the material’s behavior, particularly its response to temperature. The research demonstrates how small additions can modify a material’s characteristics without completely altering its core structure. XRD results are given in Figure 8.

The nucleation behavior of PP/pumice composites was directly impacted by the different mineralogical profiles that the XRD study showed for the three pumice sources. A greater degree of crystallinity was indicated by the noticeable reflections of mordenite, clinoptilolite–heulandite, and opal-CT in alaçatı pumice [52]. On the other hand, despite its considerable porosity, Nevşehir pumice’s vitreous amorphous phase predominated, resulting in faint crystalline signals in XRD. An intermediate nucleation potential was suggested by the mixed crystalline–amorphous structure of Kütahya pumice [52,53]. Phase changes in PP were significantly impacted by these mineralogical differences; Alaçatı pumice, in particular, showed stronger β-phase diffraction peaks, demonstrating its greater nucleating effectiveness. All things considered, these results show that the mineralogical makeup of the pumice has a major role in determining nucleation activity in PP, rather than just particle size [24].

In addition to the α-phase reflections, β-form polypropylene (β-PP) is known to exhibit characteristic diffraction peaks at 2θ ≈ 16.1°, 20.3°, and 21.8° [35,36]. In the present study, although DSC analysis was limited to α-form crystallization parameters (Tm, Tc), XRD patterns of pumice-filled composites provide indirect evidence regarding the potential presence of β-phase. Alaçatı pumice, in particular, exhibited a weak but noticeable reflection at ~16.1°, which can be associated with β-phase nucleation, consistent with its observed improvement in impact strength. In contrast, Nevşehir and Kütahya pumices did not produce a clear β-phase reflection, indicating lower β-nucleation efficiency. Therefore, XRD results suggest that regional variations in pumice mineralogy not only influence α-phase crystallization but may also promote β-phase nucleation under favorable conditions.

Although the presence of β-phase was suggested by the XRD patterns at 2θ ≈ 16.1–21.8°, the β-nucleation efficiency was not quantified in this study. Specifically, the β/α phase ratio was not calculated from peak area analysis, which represents a limitation of the present work. In the literature, the Turner–Jones method is widely applied to determine the percentage of β-phase, using the intensity ratio between the β(300) peak and the sum of the major α reflections [54]. Future studies on PP/pumice composites should therefore include quantitative β-phase analysis to more precisely evaluate the nucleating efficiency of regional pumices.

### 4.7. Scanning Electron Microscope (SEM) Examinations

**Figure 9:**
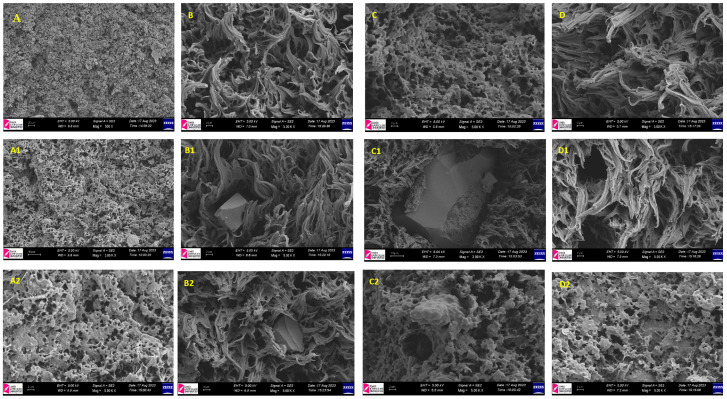
(**A**,**A1**,**A2**) PP in Different Magnification Ratios, (**B**) 0.1N, (**B1**) 0.2N, (**B2**) 0.3N, (**C**) 0.1A, (**C1**) 0.2A, (**C2**) 0.3A, (**D**) 0.1K, (**D1**) 0.2K, (**D2**) 0.3K Scanning Electron Microscope (SEM) Images.

The images from the SEM gave critical information about the microstructural changes caused by pumice in the PP matrix (Figure 9). Pumice particles were found to be evenly distributed throughout the polymer matrix with no agglomeration, according to the examination of fracture surfaces. This is important for guaranteeing uniform mechanical performance [45,46]. Particularly in samples with lower filler concentrations, the addition of pumice particles promoted heterogeneous nucleation, resulting in a more refined crystalline structure in the PP composites. Pumice particles served as nucleating sites, encouraging spherulitic development in the PP matrix, according to higher magnification SEM pictures. The source of the pumice affected the dispersion quality; Nevşehir pumice had the most consistent distribution. This is consistent with literature investigations that showed how mineral fillers help polymer matrices undergo uniform crystallization [34,35]. Nevertheless, microvoids and irregular structures were seen to form at greater pumice concentrations, which may have contributed to localized stress concentrations and decreased impact resistance [36,37]. Overall, the SEM analysis demonstrated that pumice has a significant impact on the mechanical stability and crystallization behavior of PP composites, suggesting its ability to act as an efficient nucleating agent and highlighting the necessity of the ideal filler concentration to strike a balance between processability and mechanical performance.

According to SEM micrographs, pumice’s porous structure and acidic/basic nature play a crucial role in filler–matrix interactions. At low loading (0.1 weight percent), the acidic and silica-rich alaçatı pumice showed greater interfacial adhesion with the heterophasic PP copolymer, leading to improved stress transfer and increased impact strength. However, under higher loadings, Nevşehir pumice had less bonding and interfacial voids despite being more porous, which reduced toughness. These results show that porosity and surface chemistry both affect pumice’s ability to reinforce and nucleate in PP systems.

## 5. Conclusions

This work provides the first systematic evaluation of pumice powder as a nucleating agent in polypropylene, revealing its potential to simultaneously improve toughness and thermal stability. The powder was sourced from three distinct districts of Türkiye: Nevşehir, Alaçatı, and Kütahya. Using sophisticated analytical methods like as TGA, DSC, XRD and SEM, the study concentrated on determining the mechanical and thermal characteristics of these composites. The results show that pumice successfully increases heat stability and PP’s crystallization behavior. Nevşehir pumice showed the best nucleating effect among the investigated samples, increasing HDT, Young’s modulus, and tensile strength. In contrast, the pumice samples from Alaçatı and Kütahya showed fewer homogeneous structures, which resulted in higher porosity. This, in turn, affected mechanical characteristics, including flexural strength and impact resistance.

With greater T_d_ and T_max_ values seen in all composite samples, thermal analysis verified that the addition of pumice improved PP’s resistance to thermal degradation. However, the type and concentration of pumice affected the improvement level. Higher filler loadings occasionally caused agglomeration, which impacted toughness and elasticity, but lower concentrations often produced greater impact resistance and more uniform dispersion. The deterioration of impact strength at 0.3% loading for most samples suggests that concentrations exceeding this value (e.g., 1%) would likely lead to severe agglomeration and further reduction in toughness, outweighing any potential gains in stiffness or thermal stability. Pumice appears to be a viable, low-cost, naturally occurring nucleating agent for polypropylene composites. Its use can increase mechanical performance, thermal stability, and perhaps decrease production expenses by replacing traditional fillers. All things considered, pumice shows promise as an inexpensive, environmentally friendly, and locally adjustable PP nucleating agent, with immediate ramifications for industrial uses in the consumer goods, automotive, and packaging industries. Future research should focus on two main avenues: optimizing pumice particle size and surface treatment to improve dispersion, and employing advanced characterization techniques such as isothermal crystallization kinetics (e.g., the Avrami equation) to quantitatively elucidate the nucleation efficiency and crystal growth geometry induced by the distinct pumice types.

## Figures and Tables

**Table 1:** The Technical Properties of the Pumices.

Component	Nevşehir	Alaçatı	Kütahya
Color	White	Off White-Gray	Off White-Gray
Mohs Hardness	6–6.5	6	6
Particular Weight (gr/cm^3^)	2.27	2.36	2.31
Structural Decomposition Temperature (°C)	940	916	928
Melting Point (°C)	1210	1188	1174
pH	5.5–6	5.5–6	5.5–6

**Table 2:** The Chemical Compositions of the Pumices.

Component	Nevşehir (%)	Alaçatı (%)	Kütahya (%)
SiO_2_	70.85	64.33	61.22
Al_2_O_3_	12.65	14.64	15.24
Fe_2_O_3_	1.22	3.72	2.84
Na_2_O	3.81	4.24	5.10
MgO	0.21	0.81	1.34
K_2_O	4.43	5.14	5.21
CaO	1.28	3.11	2.42
TiO_2_	0.09	0.27	0.48
A.K.	4.88	3.10	3.92

**Table 3:** Composite sample codes of PP and Pumice ratios in each composite.

Sample Codes	PP (%)	Pumice (%)
PP	100	0
0.1N	99.99	0.1
0.2N	99.98	0.2
0.3N	99.97	0.3
0.1A	99.99	0.1
0.2A	99.98	0.2
0.3A	99.97	0.3
0.1K	99.99	0.1
0.2K	99.98	0.2
0.3K	99.97	0.3

**Table 4:** Average Particle Size of P powders.

Sample	Average Particle Size (nm)	Polydispersity Index (DI)
Nevşehir	904.3	0.28
Alaçatı	549.2	0.21
Kütahya	630.5	0.25

**Table 5:** Mechanical properites of PP and its composites.

Sample Codes	Tensile Strength (MPa)	Young’s Modulus (MPa)	Flexural Strength (MPa)	Flexural Modulus (MPa)	Impact Strength (kJ/m^2^)
PP	18.05 ± 0.21	844.45 ± 38.28	21.51 ± 0.75	723.12 ± 62.83	16.51 ± 1.23
0.1N	17.39 ± 0.17	784.27 ± 64.23	21.36 ± 0.12	751.73 ± 14.37	15.46 ± 0.53
0.2N	17.99 ± 0.65	823.09 ± 56.01	21.86 ± 0.05	778.29 ± 5.42	17.14 ± 1.77
0.3N	18.99 ± 0.64	884.96 ± 53.68	22.08 ± 0.33	775.45 ± 21.27	15.26 ± 1.58
0.1A	17.38 ± 0.17	801.2 ± 8.71	22.84 ± 0.10	828.59 ± 3.95	18.30 ± 0.97
0.2A	17.08 ± 0.06	795.51 ± 5.58	21.88 ± 0.10	780.64 ± 7.49	14.76 ± 0.86
0.3A	17.27 ± 0.20	807.96 ± 15.06	21.66 ± 0.16	767.82 ± 3.44	14.27 ± 1.62
0.1K	17.63 ± 0.31	797.86 ± 5.30	22.41 ± 0.29	798.71 ± 18.96	13.68 ± 0.81
0.2K	17.7 ± 0.27	809.73 ± 23.98	22.08 ± 0.25	788.58 ± 7.82	15.25 ± 1.62
0.3K	17.97 ± 0.30	809.41 ± 37.25	22.67 ± 0.26	810.64 ± 17.39	14.87 ± 1.15

**Table 6:** TGA results of PP and Its Composites.

Sample Codes	T_d_ at 5% Mass Loss	T_max_	Mass Loss (600 °C)%
PP	402.20	456.23	98.42
0.1N	420.92	461.18	99.72
0.2N	417.98	459.06	99.11
0.3N	419.44	460.54	99.16
0.1A	421.33	460.09	99.57
0.2A	420.42	463.43	99.72
0.3A	420.50	465.05	99.07
0.1K	401.85	456.42	98.91
0.2K	420.06	459.08	99.28
0.3K	416.47	457.83	99.11

**Table 7:** DSC data of PP and Its Composites.

Sample Codes	T_c_ (°C)	ΔH_c_ (j/g)	T_m_ (°C)	ΔH_m_ (j/g)	X_c_ (%)
PP	125.23	86.72	165.51	86.02	41.16
0.1N	124.42	84.26	165.71	82.09	39.24
0.2N	124.73	84.52	165.58	82.00	39.16
0.3N	124.84	85.37	165.24	83.11	39.65
0.1A	124.67	84.44	166.04	80.52	38.49
0.2A	125.25	87.47	165.81	84.69	40.44
0.3A	124.83	86.41	166.25	82.11	39.17
0.1K	124.27	85.81	166.14	82.81	39.58
0.2K	124.60	83.66	166.28	80.31	38.35
0.3K	124.24	83.69	166.29	79.82	38.08

**Table 8:** HDT (1.8 MPa) Values of PP composite.

Sample Codes	Average Temperature (°C)
PP	45.23 ± 0.67
0.1N	47.97 ± 0.55
0.2N	48.37 ± 0.67
0.3N	48.87 ± 0.85
0.1A	46.57 ± 0.67
0.2A	46.87 ± 0.67
0.3A	46.70 ± 0.66
0.1K	48.77 ± 0.47
0.2K	48.37 ± 0.58
0.3K	48.30 ± 0.44

## Data Availability

The original contributions presented in this study are included in the article. Further inquiries can be directed to the corresponding author.
